# Optimal enteral feeding after surgery for necrotising enterocolitis: a systematic review

**DOI:** 10.1007/s00383-026-06311-y

**Published:** 2026-02-09

**Authors:** Mythili Chawan, Mehak Gupta, Tatyana Podoprigora, Iain Yardley

**Affiliations:** 1 Faculty of Medicine and Life Sciences, School of Medical Education, King’s College London GKT, Guy’s Campus, London, SE1 1UL UK; 2 Department of Paediatric Surgery, Evelina London Children’s Hospital, Guy’s and St Thomas’ NHS Foundation Trust, London, SE1 7EH UK

**Keywords:** Necrotising enterocolitis (NEC), Systematic review, Post-operative nutrition, Surgery, Clinical outcomes, Neonates

## Abstract

To review the evidence for enteral feed modification after surgery for Necrotising Enterocolitis (NEC) and determine the optimal feeding regime for these babies. A systematic review of the Scopus and PubMed databases was carried out. Papers reporting feed regimes in babies following surgery for NEC and reporting at least one clinical outcome were included. The ROBINS-I tool was utilised to evaluate the risk of bias. The search strategy returned 2772 records. After deduplication and screening against the inclusion/exclusion criteria, 11 studies were included in the review. Three studies reported the implementation of a standardised feeding regime; one led to earlier introduction of enteral feed, two found no increase in NEC recurrence and all three saw a reduction in liver dysfunction. There is a paucity of high quality evidence to guide specific post-operative feeding strategies in infants following surgery for NEC. However, limited available data suggest that achieving early enteral nutrition is not associated with worse short-term outcomes and may be associated with reduced parenteral nutrition dependence, improved growth, and lower feed-related complications. Large prospective and multicentre studies are needed to define optimal feeding composition, timing and advancement strategies to improve post-operative outcomes in this vulnerable neonatal population.

**Supplementary Information:**

The online version contains supplementary material available at 10.1007/s00383-026-06311-y.

## Introduction

Necrotising enterocolitis (NEC) is a disease affecting predominantly premature infants and is the most common emergency of the gastrointestinal tract occurring in the neonatal period [1]. It is characterised by ischaemia and inflammation of the intestine, resulting in bacterial translocation and later necrosis. Whilst there is uncertainty surrounding the causes of NEC, risk factors include low birth weight, low gestational age, sepsis, respiratory distress and formula feeds [2].

NEC can often be managed with non-operative means, including gut rest and intravenous antibiotics [3]. However, in severe cases surgical intervention is indicated, with intestinal resection and enterostomy formation being the commonest surgical approach [4, 5]. Following surgery, standard practice is to delay the re-initiation of enteral feeds for a period to allow the intestine to recover. In the meantime, parenteral nutrition (PN) is commenced and transition to enteral nutrition (EN) only occurs when deemed safe [6].

Modifications to the enteral nutrition used following surgery for NEC have been suggested, such as lipid or protein supplementation and partially or fully hydrolysed feeds [7, 8]. There is good evidence that breast milk plays a role in reducing the incidence of NEC, but its role and potential benefit to neonates following surgical NEC remains unclear [9]. It is also uncertain at what time point and how rapidly enteral feeds should be reintroduced following surgical NEC. Therefore, we performed a systematic review to identify the evidence for modifying feeds and to determine the optimal feed regime after surgery for NEC.

## Methods

*Protocol and registration*: A systematic review protocol was registered with PROSPERO (CRD42023467607).

*Data sources and search strategy*: PubMed and Scopus databases were searched on 10/10/2023 and 14/01/2026 using terms including ‘surgical’, ‘feeding’, ‘newborn’, ‘outcomes’ and MeSH terms for ‘necrotising enterocolitis’. The limitations implemented were: English language, articles from 1990-present and human studies. Table 1 summarises the search strategy used. The reference lists of systematic reviews, meta-analyses and included studies were hand-checked for other potentially missed studies.

**Table 1:** Search strategy

Databases searched	Search terms	Limits
Scopus	TITLE ( necrotic? ing AND enterocolitis ) AND ( surg* ) AND ( outcome* ) AND ( ( feed* ) OR ( nutrition* ) OR (diet* ) ) AND ( ( neonat* ) OR ( infant* ) OR ( newborn* )) AND ( LIMIT-TO ( LANGUAGE, “English” ) ) AND ( LIMIT-TO ( EXACTKEYWORD, “Human” ) OR LIMIT-TO ( EXACTKEYWORD, “Humans” ) )	English language Human studies Studies from 1990-current
PubMed	((( “Enterocolitis, Necrotizing”[Mesh])) AND ((feed*) OR (nutrition*) OR (diet*)))	English language Human studies Studies from 1990-current

*Study selection*: duplicates were removed using Mendeley, and remaining results were then title- and abstract-screened by two of three authors (MC, MG or TP) against the inclusion and exclusion criteria shown in Table 2

**Table 2:** Inclusion and exclusion criteria

Inclusion criteria	Exclusion criteria
● Neonates and infants ● Controlled trials and observational studies (case-series, case-control and cohort studies) ● Post-necrotising enterocolitis surgery ● Reporting an enteral feed intervention ● Reporting at least one outcome	● Studies not reporting surgical NEC ● Systematic reviews and meta-analyses

Full texts of remaining articles were then assessed by three authors (MC, MG, TP) against the aforementioned criteria, with inconsistencies resolved by consensus.

*Data extraction and analysis*: The data extracted from the included studies are shown in Table 3.

**Table 3:** Data extracted

Data extracted
Author
Year of publication
Study aim
Study setting
Study location
Recruitment method
Population
Inclusion and exclusion criteria
Number of participants
Age of participants at presentation
Type/mechanism of feeding
Primary outcomes (recurrence of NEC, mortality, morbidity, neurodevelopmental outcomes, duration of parenteral nutrition)
Secondary outcomes (growth measures, time to full enteral feeding, feeding complications, length of hospital stay, biochemical outcomes, e.g. cholestasis)

*Quality of included studies*: Risk of bias was assessed for all included studies using the Risk of Bias in Non-Randomised studies of interventions (ROBINS-I) tool [10]. The domains assessed were: (1) bias due to confounding, (2) bias in selection of participants into the study, (3) bias in classification of interventions, (4) bias due to deviations from intended interventions, (5) bias due to missing data, (6) bias in measurement of outcomes, (7) bias in selection of the reported result. Studies were classified as being at severe, moderate or low risk of bias, with any inconsistencies resolved by discussion.

*Data synthesis and analysis*: Where possible, data were extracted from the included articles with the intention of carrying out a meta-analysis where possible.

## Results

**Fig. 1:**
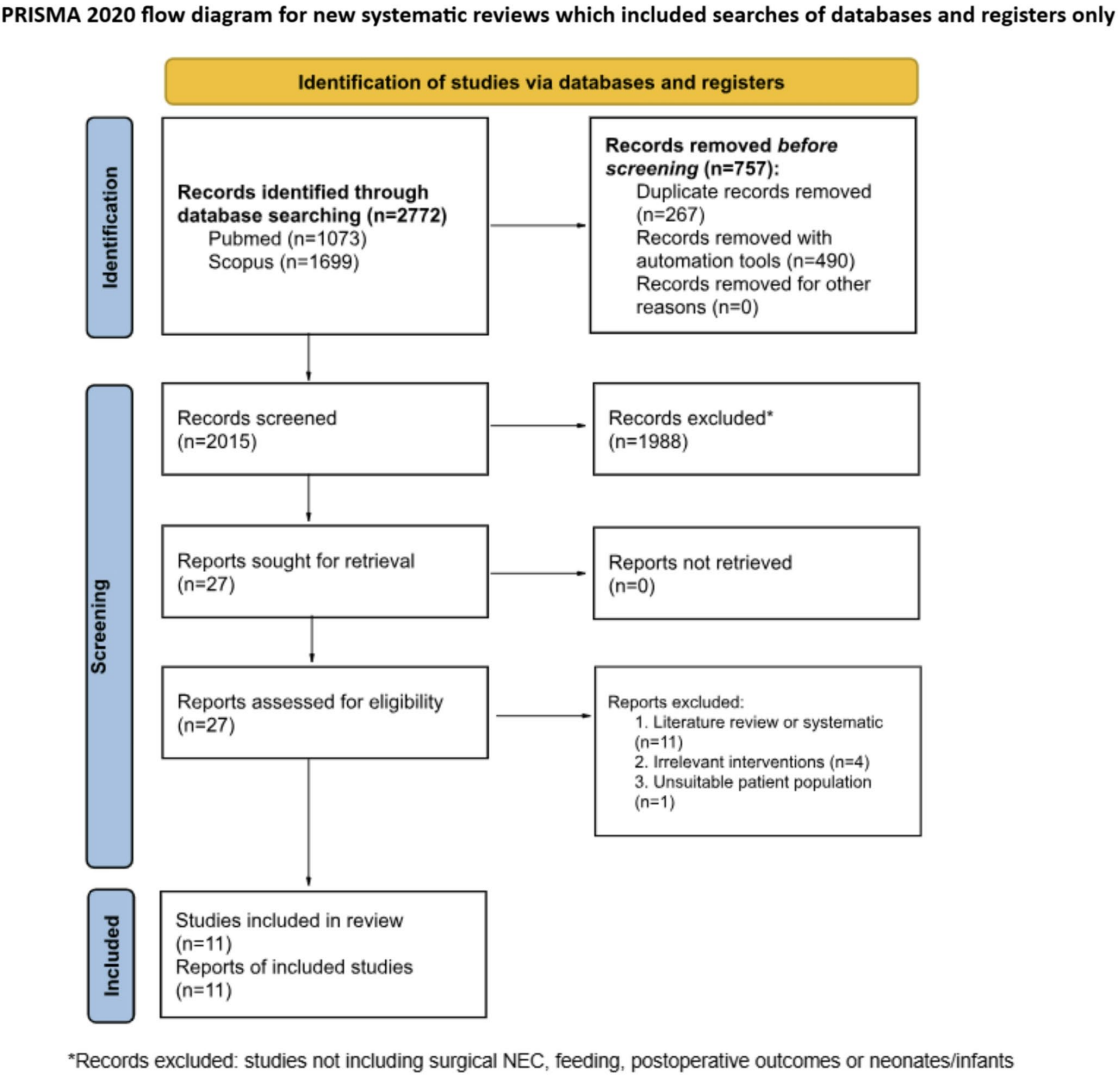
A PRISMA diagram showing the identification of studies

*Study selection*: The PRISMA flowchart (Fig. 1) details study selection and numbers included and excluded at each stage. A full record of reasons for exclusion at full-text stage can be found in Supplementary Table 1.

**Fig. 2:**
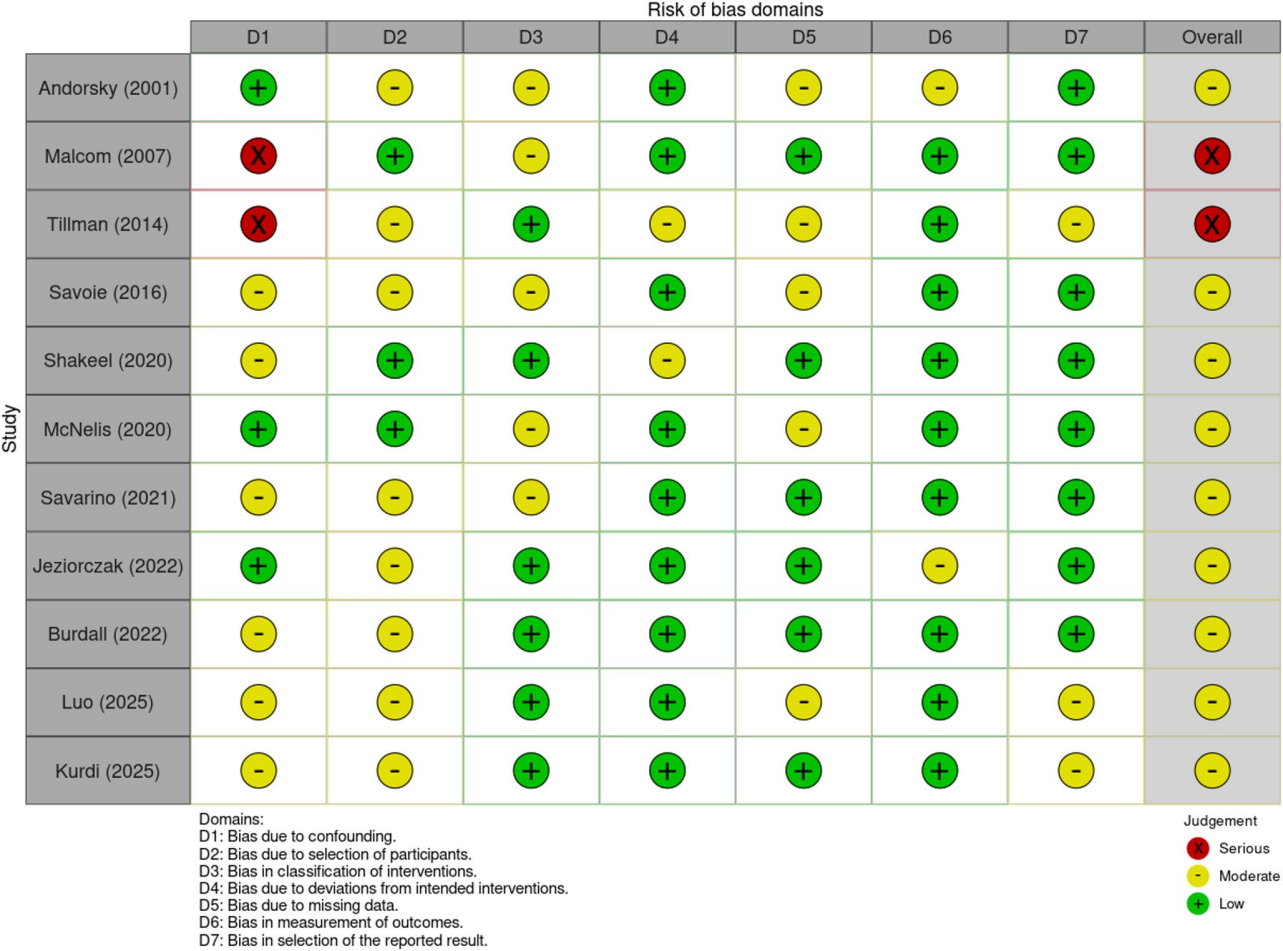
ROBINS-I table for risk of bias assessment of included articles [10]

*Quality of included studies*: A summary of the ROBINS assessment of the relevant studies can be found in Fig. 2. Malcolm et al. scored a low or moderate risk for all domains except bias due to confounding, as they were unable to attribute the increased weight gain of the infants with NEC to ‘increased calories delivered, improved calories absorbed or water weight owing to decreased ostomy losses’ [11]. The retrospective papers were more likely to have a higher risk of bias due to confounding or in the classification of interventions [12–19].

*Study characteristics*: Table 4 summarises a description of study characteristics. The papers selected consisted of seven cohort studies and one case series. All studies were retrospective, other than Shakeel et al., where the intervention cohort was prospective but historic controls were used for comparison, and Burdall et al. which is a prospective cohort study [14, 20].

**Table 4:** Summary of study characteristics

Authors	Date	Location	Study type	Number of patients	Direction of study	Interventions	Primary outcomes	Secondary outcomes
Andorsky et al. [13]	2001	United States	Cohort	13 with necrotising enterocolitis	Retrospective	Enteral feeding with breast milk and protein hydrolysed formula	Duration of PN use, peak serum direct bilirubin concentration	Successful weaning from PN, mortality, need for intestinal transplant
Malcolm et al. [11]	2007	United States	Case Series	9 with necrotising enterocolitis; 1 with isolated intestinal perforation	Retrospective	Soluble dietary fat supplement to enteral feeds	Ostomy output	Daily weight gain
Tillman et al. [19]	2014	United States	Cohort	64 with surgical necrotising enterocolitis	Retrospective	Implementation of enteral feeding guidelines	Parenteral nutrition duration, nil by mouth duration, parenteral nutrition dependence at 90 days, PNALD incidence, direct bilirubin	Weight before and after feeding guidelines
Savoie et al. [18]	2016	United States	Cohort	132*	Retrospective	Standardised feed re-introduction regime	Time to full EN after surgery	Days on PN, severity of cholestasis, breast milk use
Shakeel et al. [20]	2020	United States	Cohort	72 with necrotising enterocolitis; 67 with spontaneous intestinal perforation	Prospective with historic controls	Standardised feed re-introduction regime	Intestinal failure-associated liver disease incidence (IFALD), time to reach 50% and 100% of energy from enteral nutrition	Days on PN, postoperative NEC incidence, length of hospital stay
McNelis et al. [21]	2020	United States	Cohort	33	Retrospective	Enteral feeding advancement	Duration of PN use, differences in growth measurements (including weight, height, head circumference)	Comparative growth trajectory between surgical NEC and spontaneous intestinal perforation infants
Savarino et al. [15]	2021	Italy	Cohort	43	Retrospective	None - observational only	Survival rates of preterm infants with NEC	Time to EN after NEC onset, duration of PN, type of EN and nutritional management descriptive outcomes
Jeziorczak et al. [12]	2022	United States	Cohort	14	Retrospective	Ability to achieve enteral feeding	Survival	Bacterial infection rate, surgical intervention rate, NEC incidence, birth weight and gestational age differences
Burdall et al. [14]	2022	United Kingdom	Cohort	143	Prospective	Early (< 7 days) vs. Late Enteral Feeding	Composite of death before 28 days post-surgery, ongoing need for PN at 28 days post-surgery	Alive and off PN at 28 days, timing of feed reintroduction
Luo et al. [16]	2025	China	Cohort	90	Retrospective	Timing of enteral - early enteral nutrition (EEN, < 7 days) and late enteral nutrition (LEN, ≥ 7 days) nutrition resumption Progression of EN - faster increase (FI, ≥ 20 ml/kg/d) and slower increase (SI, < 20 ml/kg/d) Method of EN - intact protein formula, special medical formula and mixed feeding	Parenteral nutrition-associated liver disease, stoma necrosis, bowel obstruction, intestinal stenosis, reoperation	Length of stay, parenteral nutrition duration, weight gain
Kurdi et al. [17]	2025	Saudi Arabia	Cohort	500	Retrospective	Ability to achieve enteral nutrition (early (5 days), intermediate (8–14 days), and delayed (≥ 15 days) )	Early tolerance of oral intake after surgery, NEC recurrence, need to revert to PN	Feeding-related complications, validation of a predictive feeding-readiness scoring system

*66 out of 132 infants had necrotising enterocolitis

1. When is the optimal time to reinitiate enteral feeding?

Several papers emphasise the significance of achieving enteral autonomy following surgical NEC. As expected, McNelis et al. concluded that most of this population have a delay in achieving enteral autonomy, compared to neonates with medical NEC or spontaneous intestinal perforation [21]. Furthermore, Burdall et al. found no effect on mortality or need for parenteral nutrition when re-starting EN less than 7 days post-surgery [14]. Jeziorczak et al. and Savarino et al. both report that the inability to reintroduce EN after surgery for NEC is associated with higher mortality rates [12, 15]. Additionally, both papers report the potential advantages of using breast milk in EN and conclude that it does not harm babies post-surgery for NEC; however, both only report small cohorts, so the broader significance of their findings remains unclear.

Expanding on the literature for timing of initiation of EN, Luo et al. conducted a retrospective study on 90 term infants who developed perforation following NEC which required surgical intervention. It was found that earlier initiation of EN, within seven days of surgery, was significantly associated with reduced incidence of reoperation and intestinal stenosis when compared with later EN initiation. This study also reported that a faster rate of increase of EN, ≥ 20 ml/kg/day, significantly shortened PN duration and length of hospital stay, although this was associated with a slower rate of weight gain. The authors also examined types of EN feed, and it was found that infants fed with an intact protein formula had the quickest speed of advancement and no intestinal stenosis, however there were only two infants in this cohort, so generalisability is limited.

2. Are there clinical parameters that predict successful early feeding?

As retrospective or non-randomised studies, although all the above studies supported or at least found no harm from earlier feeding, it is not apparent if the babies fed sooner were systematically different from those fed later, which could account for the differences in outcomes. Kurdi et al. [17] found that early enteral feeding initiated five days after surgery was successfully tolerated in selected infants. The criteria identified as being compatible with earlier feeding included those with limited bowel resection, early stoma function and rapid normalisation of inflammatory markers, all factors that might be expected to be associated with better outcomes. The authors describe a scoring system incorporating these preoperative, intraoperative, and postoperative variables to predict feeding readiness, with no reported cases of recurrent NEC or feeding-related complications.

3. What type of milk and feeding advancement protocols are recommended post-operatively?

Two papers reported the effects of modifying the make-up of the enteral feed used following surgical NEC. Andorsky et al. included thirty neonates with short bowel syndrome, 13 following surgery for NEC, but did not segregate results according to diagnosis [13]. They found that enteral feeding with an amino acid-based formula or breast milk was associated with a reduced duration on PN and that supplementing EN with a protein hydrolysate was associated with a lower peak direct bilirubin concentration than EN alone [13]. Malcolm et al., report the effect of dietary lipid supplementation in 10 infants with enterostomies, (9 following surgery for NEC) [11]. They observed a decrease in ostomy output and an increase in daily weight gain after the addition of a soluble dietary fat supplement EN and hypothesise that this is due to the “ileal brake” phenomenon where dietary fats slow transit through the small intestine.

Shakeel et al. and Savoie et al. both report the effect of implementing standardised feeding regimes with a shared primary outcome of time to full EN [18, 20]. Both guidelines involved initiating lipid-based PN immediately after surgery, followed by EN (preferentially with breast milk) in a standardised (but different in the two studies) manner [22]. A meta-analysis of the data in these two studies was considered; however, the lack of granularity in the data and multiple different diagnoses being included made data aggregation and meta-analysis impossible. Table 5 summarises the data from these two studies.

**Table 5:** Summary of data reported in Savoie et al. [18] and Shakeel et al. [20]

Number of participants		Savoie et al. [18]			Shakeel et al. [20]					
		*n* = 132			*n* = 139					
		Control ^**a**^ (*n* = 66)	Guidelines (*n* = 66)	*P*	NEC (*n* = 73)			SIP (*n* = 67)		
					Control ^**b**^ (*n* = 49)	Guidelines (*n* = 24)	*P*	Control ^**b**^ (*n* = 38)	Guidelines (*n* = 29)	*P*
Diagnosis	NEC	33	33	1	N/A	N/A	N/A	N/A	N/A	N/A
	Gastroschisis	22	20	0.66						
	Atresia	11	13	0.62						
Outcomes	Post-operative NEC (%)	3	6	0.42	10	13	0.704	8	7	1
	Time to full enteral nutrition (days)	16	15	0.87	47	29	0.021	45	36	0.354
	Peak direct bilirubin (mg/dL)	5.6	3.9	0.01	6.5	3.5	0.019	4.4	3.5	0.391
	IFALD/PNALD (%)	55	44	0.21	80	58	0.047	73	66	0.597

*NEC* necrotising enterocolitis, *SIP* spontaneous intestinal perforation, *IFALD* intestinal failure-associated liver disease, *PNALD* parenteral nutrition-associated liver disease

^a^ Control group had no standardized feeding advancement regimen, and feeding advancement was determined based on individual physician preferences and on individual infant needs

^b^ Control group comprised a historical pre-implementation cohort managed before introduction of standardized postoperative feeding guidelines, in whom enteral feeding initiation and advancement were determined by usual clinician practice

Savoie et al. used a standardised feeding guideline that defined initiation, advancement rate and monitoring of enteral feeding after surgery for NEC [18]. Infants were stratified by residual bowel length and weight and assigned to fixed 1, 3 or 7 day advancement pathways with predetermined starting volumes and increments. Shakeel et al. implemented standardised postoperative enteral feeding guidelines for surgical infants that defined early initiation, initial feed volume and daily advancement of feeds [20]. Guidelines recommended starting feeds at 20 mL/kg/day with daily increases of 20 mL/kg/day in the absence of intolerance, with a slower alternative protocol for infants < 1000 g. Feeding tolerance criteria were predefined, and adherence was monitored using timing and volume of initial feeds and advancement rates.

Both papers report the feeding regimes being well-tolerated with no increase in post-operative NEC. Shakeel et al. reported a statistically significant reduction in time to full EN in NEC babies, but this was not seen in the Savoie study. Both studies reported potential liver protection from the standardised feeding regimes with significantly lower peak direct bilirubin levels. Furthermore, there was a lower incidence of IFALD diagnosed in all guideline groups.

Overall, Savoie et al. and Shakeel et al. both advocate for initiation of EN, with Shakeel demonstrating a significant reduction in the time taken to reach full EN. Both studies conclude that the standardised feeding regimen not only provides adequate nutrition for growth but also reduces the incidence of IFALD due to prolonged time on PN. Furthermore, both studies recognise implementing standardised clinical guidelines as feasible and encourages this to achieve improved and consistent care for patients. However, the absence of a contemporaneous control group, the lack of granular data and failure to segregate results according to diagnosis in Shakeel et al., leave the potential for several confounding factors to be at play, which may inflate apparent treatment effects and increase the risk of type I error. Alongside these factors, the small sample size also needs to be considered and the potential for type II errors that cause complications of their approach to go undetected.

Similarly to Savoie et al. and Shakeel et al., Tillman et al. investigated the impact of implementing multidisciplinary EN feeding guidelines in a retrospective before–after study of 64 infants with surgical NEC. After guideline implementation, median PN duration decreased from 106 to 65 days and median time nil by mouth from 29 to 16 days, with fewer infants remaining PN–dependent at 90 days. The incidence of PNALD declined from 73% to 42% and peak direct bilirubin concentrations were lower post-implementation. Interpretation of these findings is limited by the retrospective design, use of historical controls, small sample size, and incomplete data on parenteral nutrition exposure and residual bowel length.

## Discussion

We present a systematic review of the current literature on the optimal enteral feed regime following surgery for NEC. All included studies evaluated enteral nutrition following NEC surgery, with no evidence of harm, as evidenced by the absence of recurrent NEC in those that reported this outcome [CHANGE REFERENCE]. There is also some evidence that standardised feeding regimes can be helpful in both achieving enteral feeding and reducing IFALD and PNALD [18–20]. One study suggested earlier initiation of EN, and rapid feed advancement may mitigate long-term surgical complications, this reinforces the observation that standardised feeding regimes do not increase the risk of NEC recurrence and may improve clinical outcomes by reducing PN dependence (18). Furthermore, enteral nutrition after surgical NEC may be guided by objective radiologic, operative and postoperative recovery markers rather than fixed fasting intervals, supporting individualized early feeding strategies that may safely reduce prolonged PN, although prospective validation is required [17].

Ensuring adequate nutrition following surgery for NEC is vital to support adequate growth during a crucial period in a baby’s development. It is already known that babies living with an enterostomy are at significant risk of poor growth [23], but relying on parenteral nutrition creates the risk of liver dysfunction [24]. As babies of even lower gestational age undergo surgery for NEC [25], we can anticipate their living with enterostomies for longer periods, making enteral nutrition following surgery ever more important.

Our finding of no evidence of increased risk of recurrent NEC from enteral feeding following surgery is important and should encourage clinicians to feed babies via their gut sooner after surgery. Manipulating feed content, for example with fat supplements [11], has the potential to maximise enteral intake despite the presence of short gut or an enterostomy.

Prior efforts to systematically review the literature on enteral feeding after NEC has primarily focused on the timing of re-initiation of feeds. Patel et al. evaluated earlier versus later refeeding after NEC, demonstrating no increase in adverse outcomes with earlier initiation [26]. However, their analysis was limited to timing alone and largely included medically managed NEC. In contrast, the present review examines enteral feeding after surgical NEC as a comprehensive post-operative strategy, encompassing multiple aspects of enteral nutrition beyond timing, including feeding protocols, advancement strategies and nutritional outcomes.

Standardised feeding protocols appear to have potential to improve enteral feeding following surgery for NEC. Their use safely allows a more rapid introduction of enteral feeds and provides a framework for clinicians to refer to when managing these fragile patients. The optimum regimen however remains unclear, with differing approaches reported.

### Strengths and weaknesses

This is a thorough and reproducible systematic review with a clearly defined search strategy and a standardised screening process involving multiple researchers. We identified ten studies related to our objective and were able to retrieve full texts of all papers. A risk of bias assessment was conducted, with most articles being of low or moderate risk of bias.

However, the cohorts reported were heterogeneous, with little segregation of babies treated surgically for NEC from other diagnoses such as SIP, making the direct application of findings to surgical NEC babies unclear and an aggregated analysis impossible. In addition, surgical NEC cohorts themselves were heterogeneous, with limited differentiation between infants managed with enterostomy and those without diversion, despite these subgroups having potentially different postoperative feeding trajectories. Stratified analysis by stoma status was therefore not possible due to limited reporting in the literature and should be addressed in future studies. Almost all studies were retrospective and uncontrolled, with only two being prospective. No study included a contemporaneous comparator to their intervention, the single study that did report a control group used historical controls, making the risk of confounding significant.

### Future work

There is currently a lack of evidence to guide the enteral nutrition of infants following surgery for NEC. Further work is needed, ideally in the form of randomised or otherwise controlled trials, but if these are not felt to be feasible, then large-scale, multi-centre, prospective studies would be a useful addition. The application of standardised feeding regimes seems the most promising intervention to study. Core outcome sets for NEC [27] should be used to ensure consistent reporting across studies.

## Supplementary Information

Below is the link to the electronic supplementary material.

Supplementary Material 1

## Data Availability

No datasets were generated or analysed during the current study.
