# Supplementary material for: Optimal enteral feeding after surgery for necrotising enterocolitis: a systematic review
Source: Pediatr Surg Int. 2026 Feb 9;42(1):79. doi: 10.1007/s00383-026-06311-y (PMC12886204; doi:10.1007/s00383-026-06311-y)
Supplement: Supplementary file 1 — Supplementary Material 1 [file 383_2026_6311_MOESM1_ESM.docx]

|  | **Study** | **Reason for exclusion** |
| --- | --- | --- |
| 1 | Perks P, Borowitz S, Swanson J (2022) Nutritional management of infants with necrotizing enterocolitis. Pract Gastroenterol XLVI(1):[10-25]. | Literature review |
| 2 | Hancock J. (2000). Nursing the preterm surgical neonate. *Journal of child health care : for professionals working with children in the hospital and community*, *4*(1), 12–18. <https://doi.org/10.1177/136749350000400102> | Literature review |
| 3 | Itriago E, Trahan KF, Massieu LA, Garg PM & Premkumar MH (2023). Current Practices, Challenges, and Recommendations in Enteral Nutrition After Necrotizing Enterocolitis. *Clinics in perinatology*, *50*(3), 683–698. <https://doi.org/10.1016/j.clp.2023.04.009> | Literature review |
| 4 | Williams AF (1997) Role of feeding in the pathogenesis of necrotizing enterocolitis. Semin Neonatol 2:263-271. <https://doi.org/10.1016/S1084-2756(97)80033-4> | Expert discussion article  Discusses how feeding variables increases risk of NEC incidence, rather than how post-operative feeding affects outcomes after surgery for NEC. |
| 5 | Foglia RP (1995) Necrotizing enterocolitis. Curr Probl Surg 32(9):757-823. <https://doi.org/10.1016/S0011-3840(05)80014-0> | Literature review |
| 6 | Berlinski A, Cataltepe O (2003) The prevention and management of necrotizing enterocolitis. Curr Paediatr 13:184‑189. <https://doi.org/10.1016/S0957-5839(03)00025-3> | Literature review  Discusses the preventative strategies for NEC, as opposed to post-operative feeding affects outcomes after surgery for NEC. |
| 7 | Updegrove K (2004) Necrotizing enterocolitis: the evidence for use of human milk in prevention and treatment. J Hum Lact 20:335‑339.<https://doi.org/10.1177/0890334404266972> | Literature review |
| 8 | Good, M., Sodhi, C. P., & Hackam, D. J. (2014). Evidence-based feeding strategies before and after the development of necrotizing enterocolitis.*Expert Review of Clinical Immunology, 10*(7), 875-84. <https://doi.org/10.1586/1744666X.2014.913481> | Literature review |
| 9 | Ou, J., Courtney, C. M., Steinberger, A. E., Tecos, M. E., & Warner, B. W. (2020). Nutrition in Necrotizing Enterocolitis and Following Intestinal Resection. *Nutrients*, *12*(2), 520. <https://doi.org/10.3390/nu12020520> | Literature review |
| 10 | Lapillonne, A., Matar, M., Adleff, A., Chbihi, M., Kermorvant-Duchemin, E., & Campeotto, F. (2016). Use of extensively hydrolysed formula for refeeding neonates postnecrotising enterocolitis: a nationwide survey-based, cross-sectional study. *BMJ open*, *6*(7), e008613. <https://doi.org/10.1136/bmjopen-2015-008613> | Irrelevant intervention – looking at prevalence and reasons for using extensively hydrolysed formulas of cow’s milk proteins post-NEC surgery. |
| 11 | Pratap, A., Kaur, N., Shakya, V. C., Sapkota, G., Tanveer-ur Rahman, S., Biswas, B. K., Agrawal, C. S., & Adhikary, S. (2007). Triple tube therapy: a novel enteral feeding technique for short bowel syndrome in low-income countries. *Journal of pediatric surgery*, *42*(3), 470–473. <https://doi.org/10.1016/j.jpedsurg.2006.10.040> | 3 out of 10 infants had midgut volvulus and 7 out of the 10 included infants had NEC.  Also did not have relevant outcomes to our study. |
| 12 | Yang, M., Du, J., Yang, Q., Dou, W., Jiang, M., & Hei, M. (2021). Influence of Family Integrated Care on the Intestinal Microbiome of Preterm Infants With Necrotizing Enterocolitis and Enterostomy: A Preliminary Study. *Frontiers in pediatrics*, *9*, 678254. <https://doi.org/10.3389/fped.2021.678254> | Irrelevant intervention not related to enteral feeding modifications |
| 13 | Allin, B. S. R., Long, A. M., Gupta, A., Lakhoo, K., Knight, M., & British Association of Paediatric Surgeons Congenital Anomalies Surveillance System Necrotising Enterocolitis Collaboration (2018). One-year outcomes following surgery for necrotising enterocolitis: a UK-wide cohort study. *Archives of disease in childhood. Fetal and neonatal edition*, *103*(5), F461–F466. <https://doi.org/10.1136/archdischild-2017-313113> | Irrelevant intervention not related to enteral feeding modifications |
| 14 | Stanford, A., Upperman, J. S., Boyle, P., Schall, L., Ojimba, J. I., & Ford, H. R. (2002). Long-term follow-up of patients with necrotizing enterocolitis. *Journal of pediatric surgery*, *37*(7), 1048–1050. <https://doi.org/10.1053/jpsu.2002.33842> | Irrelevant intervention not related to enteral feeding modifications |
| 15 | Patel, E. U., Wilson, D. A., Brennan, E. A., Lesher, A. P., & Ryan, R. M. (2020). Earlier re-initiation of enteral feeding after necrotizing enterocolitis decreases recurrence or stricture: a systematic review and meta-analysis. *Journal of perinatology : official journal of the California Perinatal Association*, *40*(11), 1679–1687. <https://doi.org/10.1038/s41372-020-0722-1> | Systematic review that focuses on patients with medical necrotising enterocolitis. |
| 16 | Gosselin, K. B., & Duggan, C. (2014). Enteral nutrition in the management of pediatric intestinal failure. *The Journal of pediatrics*, *165*(6), 1085–1090. <https://doi.org/10.1016/j.jpeds.2014.08.012> | Literature review |

Supplementary table 1: Excluded papers
